# Tests and deflection calculation method for circular concrete-filled steel tubular columns under very low-elevation lateral impact loads

**DOI:** 10.1038/s41598-023-47103-x

**Published:** 2023-11-13

**Authors:** Jiayu Liang, Yanhui Liu, Yichao Zhao, Nan Xu

**Affiliations:** 1 School of Civil Engineering Southwest, Jiaotong University, Chengdu, 610031 People’s Republic of China; 2 China Construction Sixth Engineering Bureau Co., Ltd., Tianjin, 300012 People’s Republic of China

**Keywords:** Civil engineering, Nonlinear phenomena

## Abstract

An experimental investigation of circular concrete-filled steel tubular (CFST) columns subjected to very low-elevation lateral impacts was performed. Six circular CFST members were prepared for lateral impact tests according to the typical CFST columns in high-speed railway stations in China, and the impact location was at the height of the 2/9 column. The tests had three variables: the thickness of the steel tube, the impact velocity, and the axial load. The failure modes were determined in the tests, along with the time histories of the impact force and the deflection at the impact location. A finite-element analysis was performed to examine the effects of the axial load and scaling on the maximum deflection. The results show that with the increase of axial compression ratio, the impact resistance of the member first increases and then weakens. According to the travelling plastic hinge theory, a three-stage rigid plastic mechanical model was employed to describe the impact process, in which the impact location was at the non-mid-span, and a deflection calculation method for CFST applicable to any impact position was developed. A comparison with the test results indicated that deflections can be calculated with reasonable accuracy using the proposed method.

## Introduction

Concrete-filled steel tubular (CFST) structures are widely employed in civil engineering, such as high-rise buildings, bridge piers, and underground infrastructures^1–3^ due to their excellent performance^4–6^. CFST members play a significant role within the overarching structural system. These members are frequently utilized as protective elements against extreme loads, with their primary applications including vertical load-bearing columns, bridge piers, and seismic supports^1–3^. During service period, CFST structures are not only subjected to static loads but also may encounter lateral impact loads resulting from accidental or intentional incidents, such as collisions from derailed trains, ships, or aircraft^7–9^.

Compared to static loads, dynamic loads are characterized by significant forces and brief duty cycles. The impact of material nonlinearity, geometric nonlinearity, and strain rate effects on materials significantly influence the structural response. Once such accidents occur, they often result in various structural risks or problems, especially for incidents like train derailment impacts, which might lead to partial failure of the structure or even trigger the continuous collapse of the building structure, thus seriously threatening the safety of people's lives and property as well as the adverse social impacts. Therefore, understanding the dynamic response of CFST columns under these conditions can help assess their safety performance and determine their resistance and reliability in emergencies.

For the design of structures under lateral impact loads, several design codes^10–12^ have offered general rules, in which the dynamic impact loads are simplified as equivalent static forces according to the type of vehicle. Undeniably, the actual impact process is ignored in current design provisions. However, if more dynamic characteristics of the structure under impact are understood, a more accurate design can be achieved.

Experimental^13–20^ and numerical studies^13, 14, 17, 18, 21, 22^ have been performed on the behavior of CFST members under mid-span lateral impact loads since the beginning of the twenty-first century. According to extensive experiments, there are reasonable consensuses on the response characteristics associated with CFST members under mid-span lateral impact loads, which can be summarized as follows: (1) Flexural failure is observed for all CFST members^13–20^. However, the confinement factor has a significant effect on the failure behavior^17^. Members with a large confinement factor exhibit ductility under the lateral impact, whereas those with a small one generally exhibit brittleness. (2) For the members exhibiting ductility, the failure is described as the local buckling is initiated in the contact area between the impactor and the members^13, 16, 17, 20^; the global deformation begins at the mid-span of the members when the impact energy exceeds the local deformation energy; the steel tubes commonly undergo tensile fracture or rupture along the circumference; and the core concrete in the impact area is crushed under compression. (3) The impact force and the mid-span deflection are important parameters reflecting the response characteristics. Moreover, they are closely related to the impact speed, confinement factor, and boundary conditions^13–20^; however, compared with the impact force, the deflection is more significantly affected by the aforementioned factors^13^. (4) The global deformations consume most of the energy in the impact process, whereas the local deformations have low energy consumption^13, 19, 20^. (5) The axial load can influence the mid-span deflection and the impact force. When the initial impact velocity is low, the axial load can reduce the residual lateral deflection of the members, whereas when the initial velocity is high, the axial load can increase the deflection^17^. (6) The tube length does not affect the failure mode, but as the tube length increases, the local deformation increases and the impact force decreases; thus, the tube length can affect the energy distribution between the local deformation and the global deformation^13, 19^. Numerical studies on CFST members under impact loading have been conducted, such as those of Jia^13^, Wang et al.^17^, Bambach et al.^21^, Remennikov et al.^15^, Han et al.^18^, and Yousuf et al.^22^.

Moreover, for members with flexural failure, the maximum deflection is an important index of the damage levels of the member subjected to impact^23, 24^ or the explosion loads^25^ for developing guidelines for performance-based design^26^ or design procedures^14^. Furthermore, members can be simplified using mass–spring-damper models^23^. Therefore, theoretical research has been performed on calculating maximum deflection of members under lateral impacts. For CFST members, unified strength theory^2^ and the theory of travelling plastic hinges^27, 28^ have been employed to develop a calculation method for maximum deflection. For example, Jia^13^ and Qu et al.^29^ proposed a simplified deflection calculation method for CFST beams with simple supported and fixed-simple support conditions under mid-span lateral impact loads, respectively. Using Qu’s model, Shakir et al.^19^ derived a simplified deflection calculation formula for CFST members, considering the impactor's shape.

Furthermore, the axial load may affect the dynamic response of CFST columns. Research on the bending behavior of composite components is currently extensive^30–32^. Based on the available studies, the axial load ratio's influence on composite materials' impact resistance parallels its effect on their static bending strength^30^. Specifically, it has been observed that applying a certain axial force to composite components can enhance their bending performance to a certain degree^33–35^. Experimental and numerical studies^36–39^ have been performed on the behavior of CFST columns subjected to axial compression. Wang et al.^40^ proposed a simplified calculation method, considering the effect of the axial load, based on the equivalent single-degree of freedom method for predicting the deflection of axially loaded CFST members subjected to lateral impacts.

Thus far, tests on CFST members (Table 1) have mainly employed scale models. Researchers have attempted to determine the dynamic response characteristics of a large prototype by testing a small model with a similar geometry. The similarity method is governed by certain principles, which may lead to differences in the dynamic response between the small model and the large prototype, i.e., the size effect. Booth^41^ conducted 13 sets of drop hammer impact tests on sheet mild steel and structures ranging from ¼-size to full-scale and found that the dimensionless deflection of the full-scale prototype after the impact of the drop hammer was 2.5 times that of the small model, with a reduction coefficient of 0.25. Jones^28^ deduced the similarity criterion between each physical parameter in the drop hammer impact test and proposed three physical phenomena that do not conform to the similarity relation: gravity, the strain-rate sensitivity of the material, and the fracture. For the drop hammer impact test, the acceleration may reach dozens of g during impact; thus, the gravitational acceleration has little effect on the test results. However, the other two factors may lead to differences in the dynamic response between the small model and the large prototype. Jin^42^, utilizing numerical simulation methods, found that an increase in material strain rate weakens the influence of the lateral stress ratio on the size effect. Therefore, it is necessary to study the size effect.

**Table 1:** Details regarding research on the impact behavior of CFST members.

No	$D$ × $h$ × $L$ (mm × mm × mm)	Specimen number	Impact location	Boundary conditions	Researcher	Year
1	114 × (3.5–4.5) × 1200	30	0.5L	F-F/P-P/F-P	Jia^13^	2005
2	(20–50) × 1.6 × 700	6	0.5L	P-P	Bambach et al.^14^	2008
3	100 × 5 × 2500	2	0.5L	P-P	Remennikov et al.^15^	2011
4	219 × 2.9 × (1880–3150)	9	0.5L	P-P	Deng et al.^16^	2012
5	114 × (1.7–3.5) × 1200	22	0.5L	F-F/P-P/F-P	Wang et al.^17^	2013
6	100 × 5 × 2500	2	0.5L	R-P	Yousuf et al.^22^	2013
7	180 × 3.65 × (1940–2800)	9	0.5L	F-F/P-P/F-P	Han et al.^18^	2014
8	114.3 × (3–3.6) × (686–1543)	84	0.5L	F-F	Shakir et al.^19^	2016
9	114.3 × 4.5 × 1300	3	0.5L	P-P	Alam et al.^20^	2017
10	300 × 3.75 × 1300	9	0.3L	F-P	Wang et al.^46^	2017

*F-F* fixed–fixed, *P-P* pinned–pinned, *F-P* fixed–pinned, *R-P* rolled–pinned.

In recent years, with the development of high-speed railway and road transportation, the threat of vehicle impacts on structures has increased, prompting some researchers^43–46^ to focus on asymmetric impacts (where the impact location is not at the mid-span). However, compared with symmetric impacts, the dynamic response of CFST members under asymmetric impacts still needs to be adequately understood. A recent numerical study by Alam^43^, indicated that CFST columns exhibit flexible behavior with large global deformations at mid-height zones under the vehicular impact, which differs significantly from the results of the numerical simulations of Jia^13^ and Wang’s test study^46^. In Jia’s study^13^, the maximum deflection of the CFST members occurred at the impact location. Wang performed a series of tests on circular CFST columns under lateral impact loads^46^. The impact location was 1/3H, the typical position of a vehicle (truck) impact, and the test results showed that the CFST columns tended toward shear failure.

Detailed information regarding all the tests is presented in Table 1. In the boundary condition of the table, F stands for fixed, P stands for pinned, and R stands for rolled.

**Figure 1:**
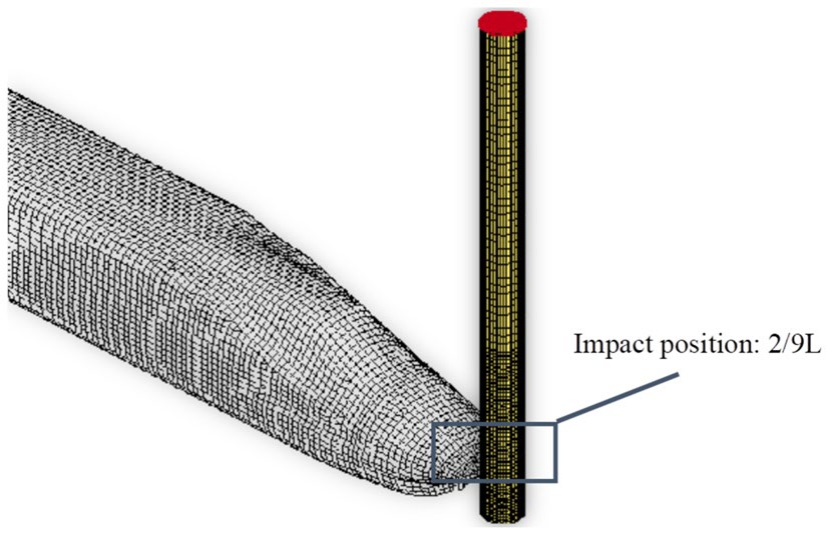
Schematic of the train impact column.

At present, systematic research on the influence of asymmetry on CFST members remains limited. Kang^47^ has developed an alternative modeling approach that effectively predicts the response of CFST columns under low-velocity impact loads. Although the maximum deflection is considered as an important index for CFST members under lateral impact, no universal formula has been proposed for calculating the maximum deflection at any impact location for members. Therefore, this study aims to employ experimental and numerical methods to study the performance of CFST columns subjected to very low-elevation lateral impact, which is the lateral impact load applied near the base of the column. Six CFST members were designed based on the typical CFST columns in high-speed railway stations in China, and the impact location was determined according to the contact point between the running train and the column, as shown in Fig. 1. The characteristics of the impact are that the impactor carries large energy, the boundary condition is fixed at both ends, the impact point is close to the column base, and the dynamic response performance of the circular CFST station column under the very low height lateral impact is focused. The objectives of this paper were as follows: (1) to investigate the dynamic response of the members, including the failure mode, time-history curves of the impact force and deflection, and the relationship between the impact force and the deflection; (2) to investigate the influence of the axial load and scaling on the maximum deflection via finite-element analysis (FEA); and (3) to develop a rigid-plastic mechanical model for establishing a theoretical deflection calculation method that can be generally applied to CFST columns at any impact location.

## Test

### Material properties

#### Steel tubes

The steel tubes used in the test were fabricated using Chinese Standard Q235 steel. The Young’s modulus (*E*_s_) and the yield strength (*f*_y_) were tested using a 500KN microcomputer-controlled universal test machine in accordance with the Chinese Standard for metallic materials (GBT228-2002)^48^. The test results for the two tensile coupons are presented in Table 2.

**Table 2:** Properties of the steel tubes.

Test coupons	Young’s modulus *E*_s_ (GPa)	Yield strength *f*_y_ (MPa)	Ultimate strength *f*_u_ (MPa)	Elongation (%)
2.0 mm steel tube	198	338	377	21.68
3.5 mm steel tube	223	323	393	21.57

#### Concrete

Nine 150 × 150 × 150 mm^3^ concrete cubes, which were cast and cured under the same conditions as the concrete used in the impact tests, were prepared to determine the concrete compressive strength of the specimens. The concrete, used as the filling material, and the nine concrete cubes were carried out under the same conditions, all cured in a humid environment. The concrete strength class was C40, and the average cubic compressive strength was 54.97MPa. The test results for the nine test coupons are presented in Table 3.

**Table 3:** Properties of the concrete.

Test coupons	1	2	3	4	5	6	7	8	9	Average
Concrete cube compressive strength *f*_cu_ (MPa)	56.11	54.29	50.96	54.72	57.11	52.24	57.51	53.59	58.21	54.97

#### Test members and setup

**Figure 2:**
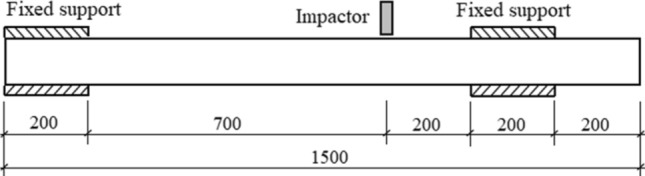
Member details and dimensions (unit: mm).

To investigate the dynamic response of the CFST members under a non-mid-span impact, lateral impact tests were conducted using self-weight drop hammer impact testing equipment. Six sets of CFST specimens were designed and fabricated at a scale of 1:10, according to the typical CFST columns in the high-speed railway stations of China. Jones studied the scaling criteria of various parameters in the drop hammer impact test^28^. Table 4 presents the ratio of the physical parameters of the prototype to those of the model when the geometric ratio of the prototype to the scaled model was η (in the tests, η was 0.1). The ratios of the parameters shown in Table 4 were used to design the tests. Details regarding the specimens are presented in Table 5. The total length of the specimens was 1.5 m, with a clear span of 0.9 m. The impact position for all the specimens was at 2/9 of the span, and the dimensions of the test segments are presented in Fig. 2.

**Table 4:** Parameters in the prototype model relative to the reduced model.

Parameter	Ratio	Parameter	Ratio
Geometric dimension	η	Acceleration	η^−1^
Mass	η^3^	Time	η
Material property	1	Force	η^2^
Velocity	1	Stress	1

**Table 5:** Details of the test specimens.

Specimen label	*M* (kg)	*V*_0_ (m/s)	*l*_1_ (m)	*l*_2_ (m)	*m* (kg/m)	*D* (mm)	*h* (mm)	*F* (kN)
YG1	270	7.67	0.2	0.7	31.3	114	2	0
YG2	270	9.90	0.2	0.7	31.3	114	2	0
YG3	270	11.71	0.2	0.7	31.3	114	2	0
YG4	270	11.71	0.2	0.7	32.1	114	3.5	0
TS1	270	9.90	0.2	0.7	32.1	114	3.5	0
YG7	270	9.90	0.2	0.7	32.1	114	3.5	200

#### Test apparatus

**Figure 3:**
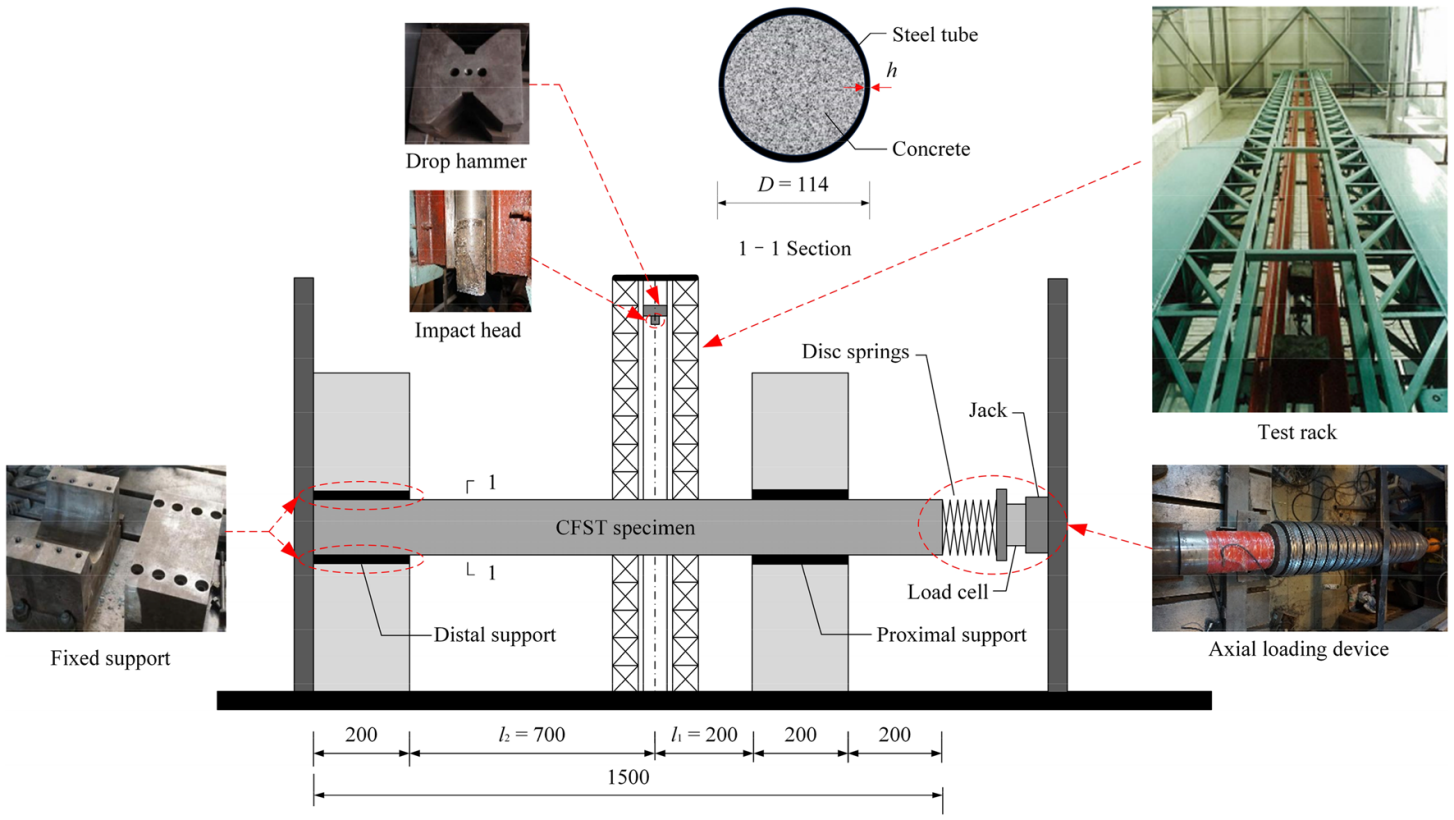
Drop hammer impact test device diagram.

The lateral impact tests were performed using the DHR9401 drop hammer impact testing equipment. This equipment comprised the following segments: a test frame, a drop hammer, an impact head, supports at both ends, a load cell, and other ancillary equipment, as shown in Fig. 3. The test frame was composed of a concrete foundation, rigid beams and two steel columns. The test impact head, whose bottom surface was a rectangle with dimensions of 30 × 80 mm^2^ and whose top surface was a circle with a diameter of 80 mm, was made of chromium 15 with 64HRC hardness. The boundary conditions were kept as a fixed support utilizing four different segments: the upper and lower half-rings, which were connected and fixed by bolts. An axial load loading device was equipped based on the drop hammer impact device. An axial load loading device is installed behind the proximal support. The unit comprises a disc spring pack with a 500 kN axial hydraulic jack and a load cell between the disc spring set and the jack. A hydraulic jack applies the axial load on the specimen through the load cell.

In the test, the member was placed on the impact table, then an axial force load was applied to it at the end of the member, and finally, the drop hammer was lifted to the design height and then released to impose the impact load. A high-speed camera (HSC) was employed to capture the failure modes of the specimens. The impact force was recorded by using the load cell built into the drop hammer.

### Test results and discussions

#### Failure modes

**Figure 4:**
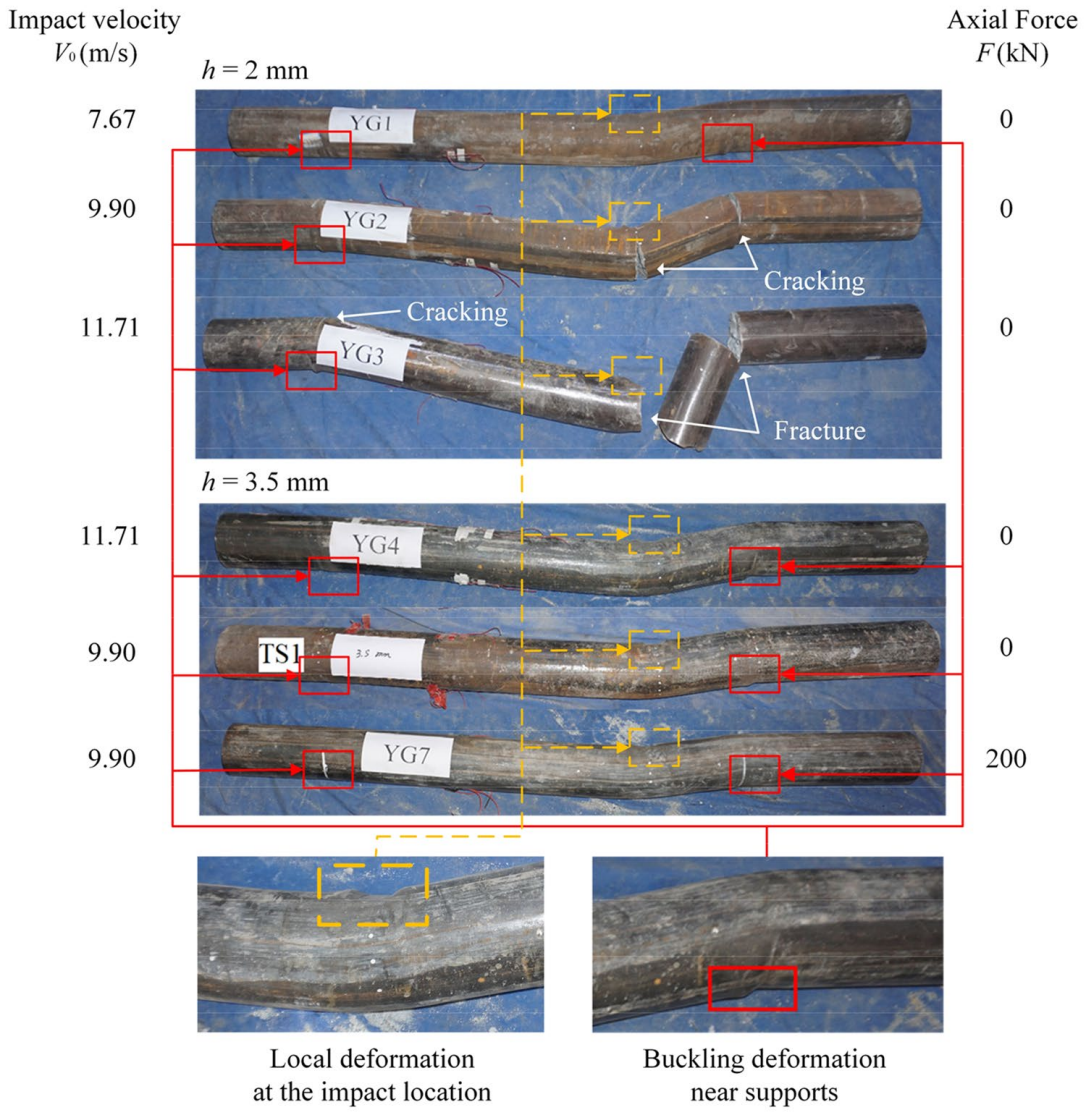
Failure modes of the tested members.

The final failure modes of the specimens were identified by analyzing the whole impact process, and the global deformation of the specimens is shown in Fig. 4. Under the influence of a 2/9 cross-lateral impact, it was found that specimens, regardless of impact velocity, thickness of steel tube, and axial pressure, predominantly experienced bending failure. This bending was often accompanied by cracks or fractures, with the peak deflection of the CFST member occurring at the impact site. Furthermore, flexural deformation occurred, but no cracks appeared in YG1, TS1, YG4, and YG7. Flexural cracks perpendicular to the axial direction of the members were observed in YG2. Although YG3 was completely fractured, it exhibited flexural cracks. Figure 4 further revealed varying degrees of local buckling at both the impact site and the supports at each end. For a lateral impact condition corresponding to a 2/9 net span, the specimen's deformation differed from the symmetrical deformation observed under cross-medium impact. Relative to the deformation at the distal support area, deformation was more pronounced in the impact position and proximal support areas. Consequently, a more pronounced plastic hinge manifested in these regions.

#### Impact force

**Figure 5:**
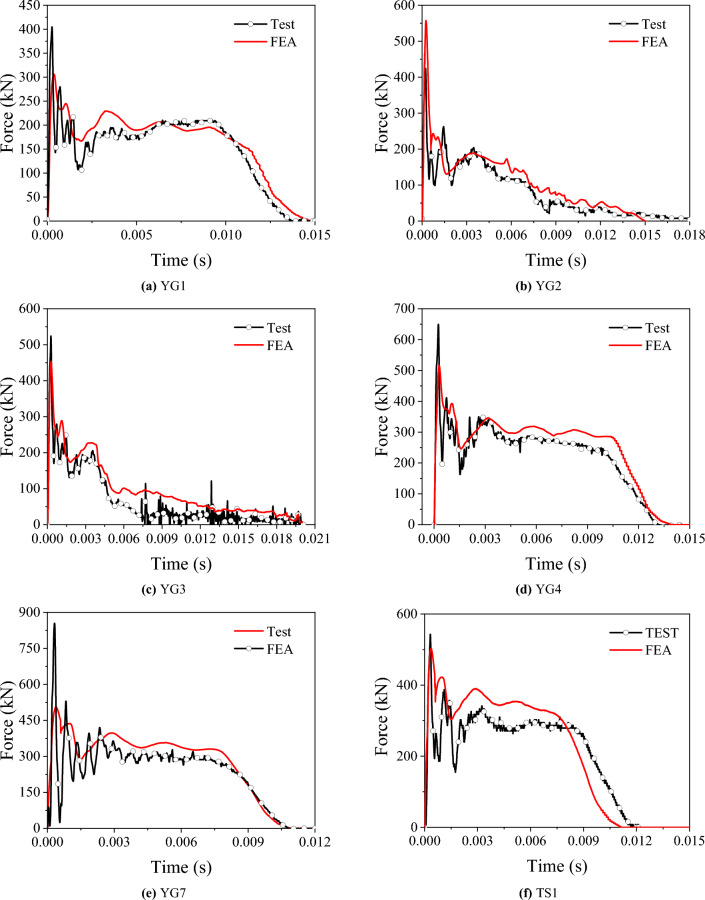
Impact force–time curves.

The impact force-time history curve of the test is shown in Fig. 5, after the impact process starts, the impact force increases to the peak in a very short time. Subsequently, the deformation speed of the specimen gradually decreases, and the impact force of YG1, YG4, TS1 and YG7 enters a stable state, meaning it stabilizes at the impact force platform value. Then, the impactor rebounds and the impact force gradually decreases to 0. The impact force of YG2 and YG3 enters the descending phase after reaching the peak, resulting from YG2 cracking during the impact process and YG3 fracturing.

#### Deflection

**Figure 6:**
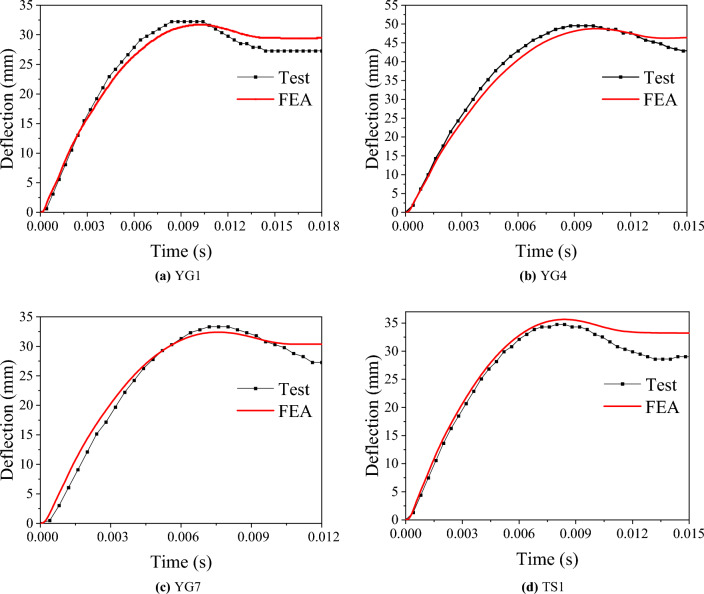
Deflection-time curves.

The deflection-time history curve of the specimen can be seen in Fig. 6. The results show that the deflection of the unbroken specimen increases continuously after the impactor hits the specimen. As the impactor’s velocity gradually decreases to 0, the deformation velocity of the specimen gradually decreases to 0. At this moment, the deflection reaches the maximum value. Then, the impactor rebounds upward, and the deflection of the specimen decreases to a certain extent and finally tends to flatten.

#### Energy absorption

**Figure 7:**
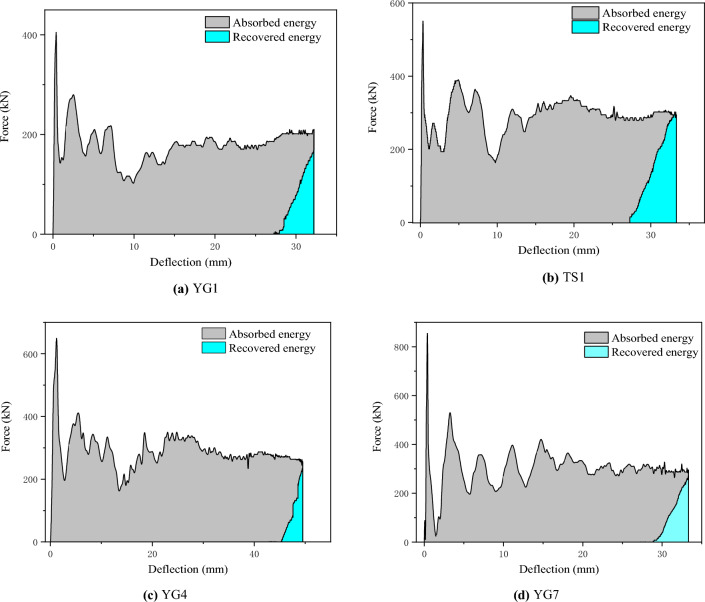
Force–deflection curve.

In the tests, the deflection-time curves were obtained using the HSC, and the force-time curves were obtained using a load cell built into the drop hammer. The results for the lateral force and lateral deflection are presented in Table 6. The force-time and deflection-time curves are shown in Figs. 5 and 6, respectively. The area beneath the force-displacement curve was used to calculate the recovered and absorbed energy, as shown in Fig. 7. The energy absorption ratio to the total energy applied was then determined, as shown in Table 6.

**Table 6:** Test results.

Specimen label	$\Delta$(mm)	Maximum force (kN)	Absorbed energy (J)	Recovered energy (J)	Absorbed energy ratio
YG1	32.2	410.9	5432	367	93.7%
YG2	Fracture	449.6	/	/	/
YG3	Fracture	527.1	/	/	/
TS1	34.8	542.6	9204	937	92.4%
YG4	49.5	658.9	13,950	422	97.1%
YG7	33.3	852.7	10,100	538	94.7%

Absorbed energy ratio = absorbed energy/(absorbed energy + recovered energy).

The test results demonstrate that increasing the initial impact energy (by increasing the impact velocity while keeping the impact mass constant) increases the overall deformation energy consumption of the specimen to varying degrees. The plastic deformation of the specimen will be correspondingly aggravated, weakening the specimen's elastic deformation recovery degree. Then, the rebound kinetic energy of the impactor will decrease accordingly.

### Finite-element analysis

#### Finite-element model

**Figure 8:**
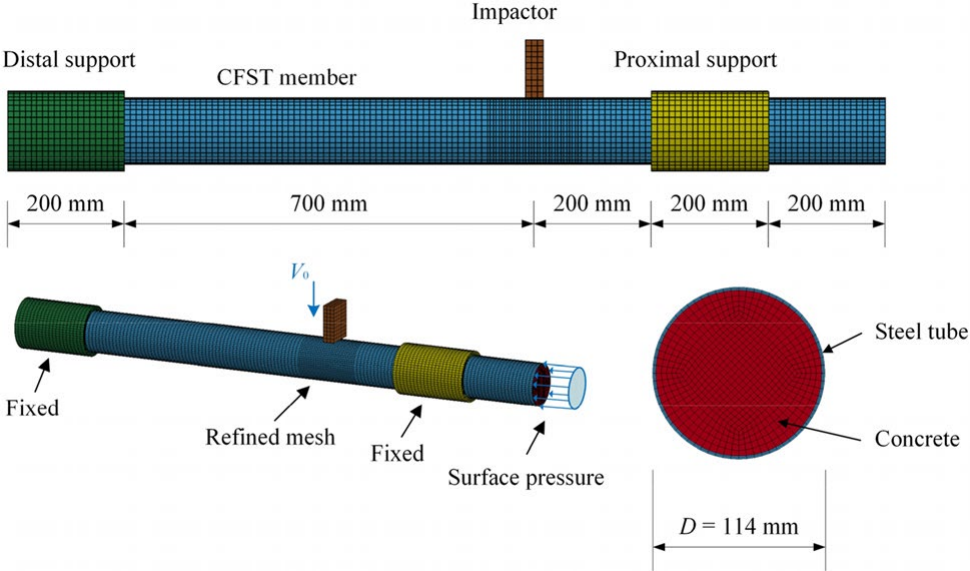
FEA model of the CFST member.

The commercial finite-element program LS-DYNA^49^ was used to simulate the drop-weight impact test. Figure 8 shows the FEA model of the CFST member under the drop hammer impact. The drop hammer, steel tube, and core concrete were simulated using 8-node solid elements with reduced integration.

Regarding the material parameters, the properties of the concrete followed the concrete damage model, and the strain-rate effect of the concrete was considered according to the dynamic increase factor (DIF). The DIF for the compressive strength of concrete was defined by Malvar et al.^50^ as follows:

1 $${\text{DIF}} = \frac{{f_{{{\text{cd}}}} }}{{f_{{{\text{cs}}}} }} = \left\{ \begin{gathered} (\dot{\varepsilon }/\dot{\varepsilon }_{{\text{s}}} )^{{1.026\alpha_{{\text{s}}} }} ,\dot{\varepsilon } \le 30{\text{s}}^{ - 1} \hfill \\ \gamma (\dot{\varepsilon }/\dot{\varepsilon }_{{\text{s}}} )^{1/3} ,\dot{\varepsilon } > 30{\text{s}}^{ - 1} \hfill \\ \end{gathered} \right.,$$

where *f*_cd_ represents the dynamic compressive strength of the concrete at the strain rate $\dot{\varepsilon }$; *f*_cs_ represents the static compressive strength of the concrete at the strain rate $\dot{\varepsilon }_{s}$ = 30 × 10^–6^; logγ = 6.156α_s_ − 2; α_s_ = (5 + 9*f*_cs_ */ f*_co_)^−1^; and *f*_co_ = 10 MPa. The DIF for the tensile strength of concrete is given as

2 $${\text{DIF}} = \frac{{f_{{{\text{td}}}} }}{{f_{{{\text{ts}}}} }} = \left\{ \begin{gathered} (\dot{\varepsilon }/\dot{\varepsilon }_{{\text{s}}} )^{{1.016\delta_{{\text{s}}} }} ,\dot{\varepsilon } \le 30{\text{s}}^{ - 1} \hfill \\ \beta (\dot{\varepsilon }/\dot{\varepsilon }_{{\text{s}}} )^{1/3} ,\dot{\varepsilon } > 30{\text{s}}^{ - 1} \hfill \\ \end{gathered} \right.$$

where *f*_td_ represents the dynamic tensile strength of the concrete at the strain rate $\dot{\varepsilon }$; *f*_ts_ represents the static tensile strength of the concrete at the strain rate $\dot{\varepsilon }_{s}$ = 30 × 10^–6^; logβ = 7.11δ_s_ – 2.33; and δ_s_  = (10 + 6*f*_cs_ */ f*_co_)^−1^. To simulate the failure of the concrete, the failure strain of the concrete was defined by MAT_ADD_EROSION in LS-DYNA. Malvar and Crawford proposed a DIF formulation for steel, with the yielding stress varying from 290 to 710 MPa^51^:

3 $${\text{DIF}} = \frac{{f_{yd} }}{{f_{ys} }} = (\dot{\varepsilon }/10^{ - 4} )^{\alpha }$$

where *f*_yd_ represents the dynamic yield strength of the steel at the strain rate $\dot{\varepsilon }$, *f*_ys_ represents the static yield strength of the steel, and α = 0.074 – 0.040*f*_y_ / 414.

The impactor and the supports were regarded as rigid bodies without considering the influence of deformation. For the impactor and the supports, a rigid model was used. The keyword CONTACT_AUTOMATIC _SURFACE_TO_SURFACE was utilized to simulate the contact between the impactor and the specimen and that between the specimen and the support. The contact between the steel tube and concrete elements was achieved using co-node methods, and the relative slip was ignored. The axial load was applied to the transverse section as a uniform surface pressure, and the axial load was defined by DEFINE_CURVE, with its position and direction defined by LOAD_NODE_SET.

### Comparison of simulation and test results

**Figure 9:**
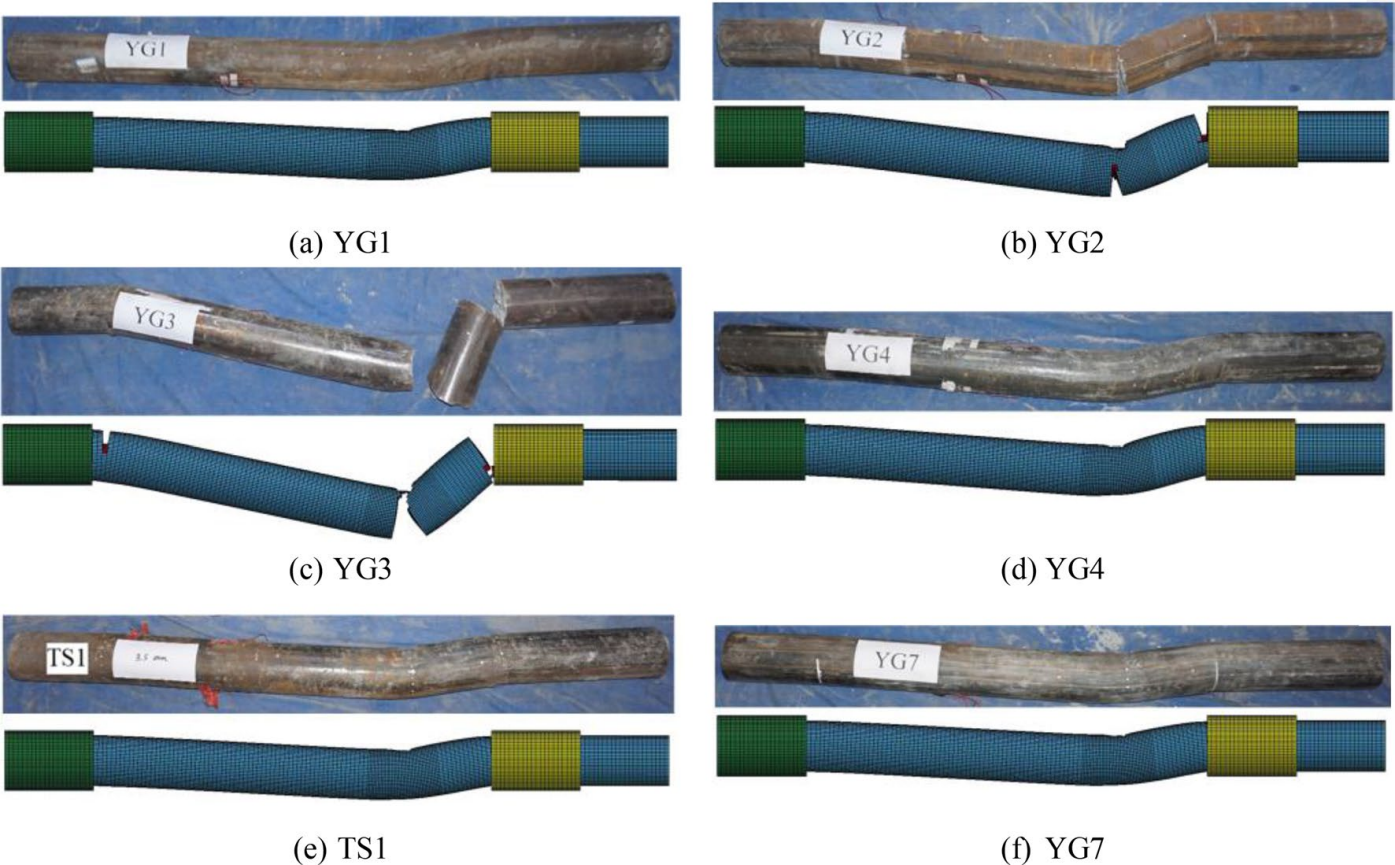
Comparison of the failure modes of the numerical model and the test.

The failure modes, the impact force-time curves and deflection-time curves are shown in Figs. 5, 6 and 9, respectively, and the accuracy of the parameters used in the numerical simulation was verified via comparison with the test results. From the comparison of failure modes shown in Fig. 9, the finite element model can reasonably simulate specimens' deformation and failure modes. The trends of the impact-force and displacement time history curves were similar for the two groups of specimens. Detailed comparison results, including the maximum deflection, plateau value of the impact force, and total duration, are presented in Table 7. Overall, the test and simulation results agreed well, and the errors were within a reasonable range, confirming the sufficient accuracy and precision of the parameters used in the simulation.

**Table 7:** Comparison between the simulation and test results.

Specimen	Maximum deflection (mm)			Plateau values of the impact forces (kN)			Total duration (s)		
	Test	FEA	Error	Test	FEA	Error (%)	Test	FEA	Error
YG1	32.2	31.1	–3.40%	209	223	6.70	0.0138	0.0133	–3.60%
YG2	/	/	/	186	170	–8.60	/	/	/
YG3	/	/	/	202	186	–7.92	/	/	/
YG4	49.5	48.7	–1.62%	286	294	2.80	0.0131	0.0139	6.11%
YG7	33.3	32.4	–2.70%	288	320	11.1	0.0108	0.0109	0.93%
TS1	34.8	35.6	2.30%	303	328	8.25	0.0116	0.0111	–4.31%

### Effect of axial load on deflection

**Figure 10:**
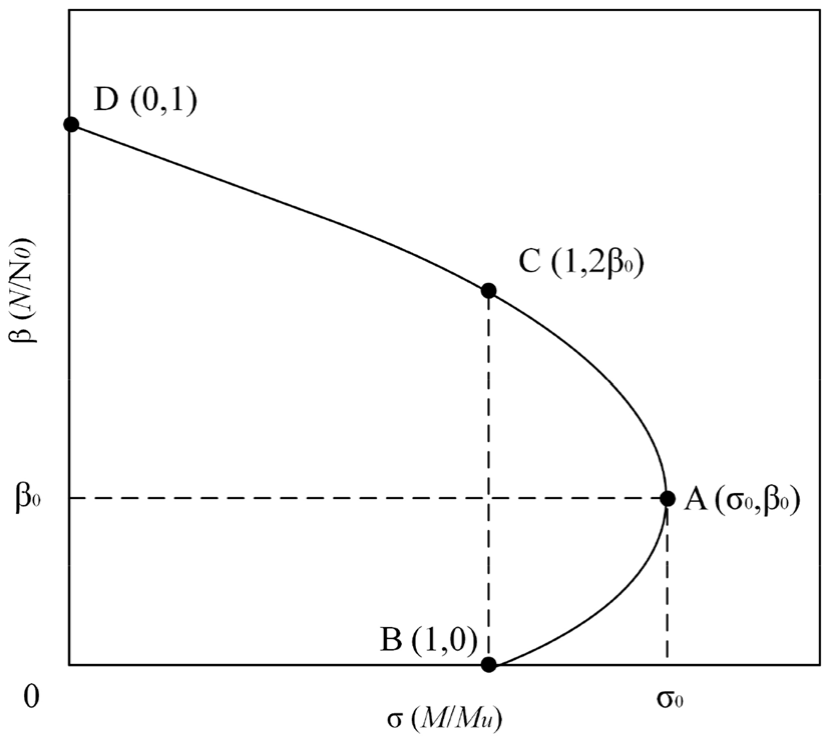
Axial load versus moment interaction curves.

Under the influence of lateral load, CFST members primarily undergo bending failure. A curve is illustrated in Fig. 10, which depicts the *F*/*F*_o_-*M*_s_/*M*_u_ curve for a typical CFST member^30^. In this curve, the x-axis represents the ratio of the section bending moment value *M*_s_ to the bending bearing capacity *M*_u_, and the y-axis represents the ratio of the axial force *F* of the section to the axial compressive strength bearing capacity *F*_o_ (i.e., axial compression ratio *n*). From Fig. 10, it is evident that when the axial compression ratio *n*<2β_0_, the bending moment value of the section is greater than the bending bearing capacity, indicating that the existence of axial force improves the bending resistance of CFST. When the axial compression ratio *n*>2β_0_, the bending moment value of the member section is continuously reduced with the increase of axial force, and the axial force reduces the bending performance of the member.

**Figure 11:**
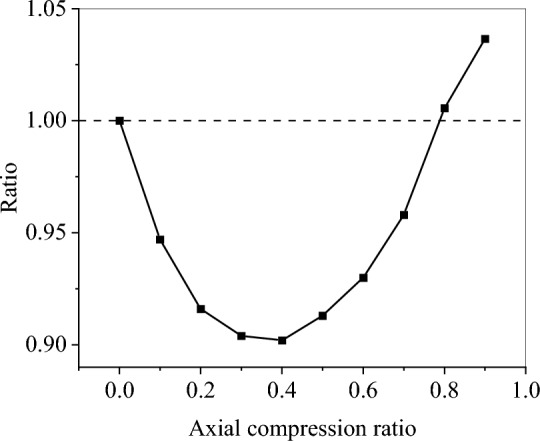
Relationship between the deflection and the axial compression ratio.

The test parameters of specimens TS1 and YG7 were identical, except for the axial load. The maximum deflections of the two specimens were 34.8 and 33.3 mm, respectively (a slight difference of 4.3%). This phenomenon indicates that the axial load only slightly affected the deflection of the unbroken member. To describe the effect of the axial load more accurately, the impact processes of TS1 components under different axial compression ratios were supplemented using LS-DYNA software (Table 8). As shown in Fig. 11, with an increase in the axial compression ratio, the maximum deflection of the CFST members first decreased and then increased. When the axial compression ratio was approximately 0.4, the deflection was minimized. When the axial compression ratio was approximately 0.8, the deflection was close to that without an axial load. When the axial pressure was <0.8, the axial load improved the lateral impact resistance of the CFST members. When the axial compression ratio was >0.8, the axial load reduced the lateral impact resistance of the CFST members. Under different axial compression ratios, the maximum deflections of the members were close (within 10%).

**Table 8:** Effect of the axial compression ratio on the deflection.

Axial compression ratio	0	0.1	0.2	0.3	0.4	0.5	0.6	0.7	0.8	0.9
Maximum deflection	35.6	33.7	32.6	32.2	32.1	32.5	33.1	34.1	35.8	36.9
Error	0	− 5.3%	− 8.4%	− 9.6%	− 9.8%	− 8.7%	− 7.0%	− 4.2%	0.56%	3.65%

### Scaling effect on deflection

The response of CFST members under the impact of the lateral drop hammer involves multiple physical quantities, and the relationships between these physical quantities can be determined via dimensional analysis. The physical quantities that determine the maximum deflection *w*_0_ mainly include the

1. Impactor parameters, i.e., impactor mass *M*, density *ρ*_I_, impact surface area *S*, and impact velocity *V*_0_;
2. Concrete material properties, i.e., density *ρ*_c_, elastic modulus *E*_c_, compressive strength *f*_c_, and Poisson's ratio *μ*_c_;
3. Material properties of steel, i.e., density* ρ*_s_, elastic modulus *E*_s_, yield strength *f*_y_, and Poisson's ratio *μ*_s_; and
4. Component geometry, i.e., outer diameter of the circular steel tube *D*, thickness of the steel tube *h*, distance *l*_1_ from the impact point to the left support, and distance *l*_2_ from the impact point to the right support.

According to the foregoing 16 physical quantity parameters, the functional relationship between the maximum deflection and each physical quantity can be expressed as follows:

4 $$w_{0} = f(M,\rho_{{\text{I}}} ,S,V_{0} ,\rho_{{\text{c}}} ,E_{{\text{c}}} ,f_{{\text{c}}} ,\mu_{{\text{c}}} ,\rho_{{\text{s}}} ,E_{{\text{s}}} ,f_{{\text{y}}} ,\mu_{{\text{s}}} ,h,D,l_{1} ,l_{2} )$$

Three physical parameters with independent dimensions—the cross-sectional diameter *D* of the member, the elastic modulus of the steel *E*_s_, and the density of the steel *ρ*_s_—are selected as the basic quantities. Equation (5) can be derived from Eq. (4) based on the π theorem:

5 $$\begin{gathered} \frac{{w_{0} }}{D} = a_{0} \left( {\frac{M}{{\rho_{{\text{s}}} D^{3} }}} \right)^{{a_{1} }} \left( {\frac{{\rho_{{\text{I}}} }}{{\rho_{{\text{s}}} }}} \right)^{{a_{2} }} \left( {\frac{S}{{D^{2} }}} \right)^{{a_{3} }} \left( {V_{0} \sqrt {\frac{{\rho_{{\text{s}}} }}{{E_{{\text{s}}} }}} } \right)^{{a_{4} }} \left( {\frac{{\rho_{{\text{c}}} }}{{\rho_{{\text{s}}} }}} \right)^{{a_{5} }} \left( {\frac{{E_{{\text{c}}} }}{{E_{{\text{s}}} }}} \right)^{{a_{6} }} \hfill \\ \left( {\frac{{f_{{\text{c}}} }}{{E_{{\text{s}}} }}} \right)^{{a_{7} }} \left( {\mu_{{\text{c}}} } \right)^{{a_{8} }} \left( {\frac{{f_{{\text{y}}} }}{{E_{{\text{s}}} }}} \right)^{{a_{9} }} \left( {\mu_{{\text{s}}} } \right)^{{a_{10} }} \left( \frac{h}{D} \right)^{{a_{11} }} \left( {\frac{{l_{1} }}{D}} \right)^{{a_{12} }} \left( {\frac{{l_{2} }}{D}} \right)^{{a_{13} }} \hfill \\ \end{gathered}$$

where *a*_0_–*a*_13_ are real numbers to be determined.

Equation (5) provides a general calculation model for such problems. It is suitable for establishing empirical models for maximum deflection calculation with different steel and concrete materials, geometric parameters, and impact conditions.

When the parameters in the model and the prototype are set to the values presented in Table 4, the relationship between the model and the prototype can be expressed as

6 $$\left( {\frac{{w_{0} }}{D}} \right)_{{\text{m}}} = \left( {\frac{{w_{0} }}{D}} \right)_{{\text{p}}}$$

where the subscripts m and p represent the model (small-scale experiment) and the prototype (full-scale experiment), respectively.

**Figure 12:**
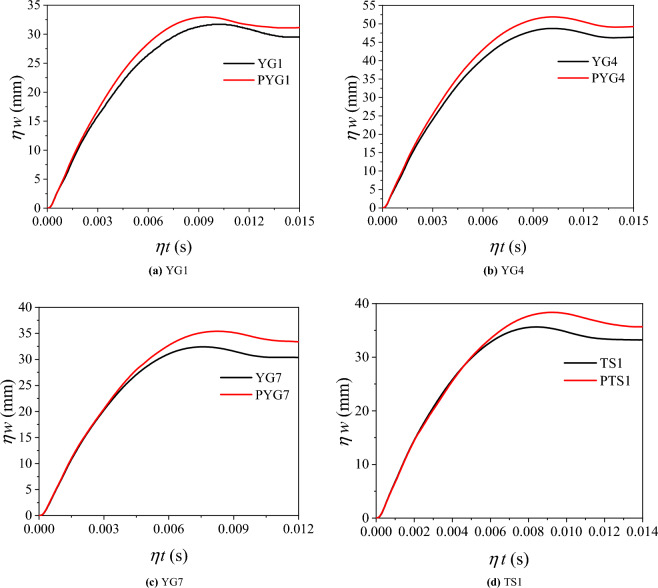
*ηw − ηt* curves.

Equation (6) indicates that when parameters are set in strict accordance with the similarity theory, as shown in Table 4, the deflection of the components in the scaled CFST drop weight impact test conforms with the similarity criterion. To further verify the similarity, an FEA model of each component in the experiment and one of the full-scale prototype components were established. Then, *ηw−ηt* curves obtained from finite-element calculations were compared (*η = 0.1* for the prototype component and *η = 1* for the model component), as shown in Fig. 12. The deflection variations of the prototype and the model exhibited the same trend, confirming that the deflection of the component satisfied the assumption of the similarity theory. Table 9 shows that the deflection error between the scaled-down model and the full-scale prototype falls within 7%. Consequently, the size effect was ignored in this experiment.

**Table 9:** Size effect on the deflection.

Specimen	YG1	PYG1	YG4	PYG4	YG7	PYG7	TS1	PTS1
*ηw*_0_(mm)	31.1	32.8	48.7	51.9	32.4	32.7	32.7	32.5
Error	5.47%		6.57%		0.93%		− 0.61%	

### Velocity variation at impact point

**Figure 13:**
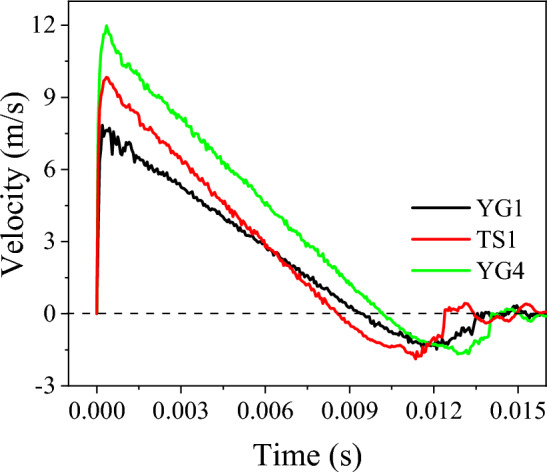
Impact velocity history from LS-DYNA.

The lateral velocity variation at the impact point of the specimens, caused by the lateral impact, is shown in Fig. 13. At the beginning of the impact, the velocity increased sharply to the maximum value in a very short time. Then, the velocity decreased nearly linearly with respect to time. Because the impact force was essentially stable after a short fluctuation in the initial stage of impact (Fig. 5), its acceleration was also stable, eventually leading to a linear decrease in the velocity over time. When the velocity decreased to zero, the specimen reached the maximum deflection.

### Moment variation at impact point

**Figure 14:**
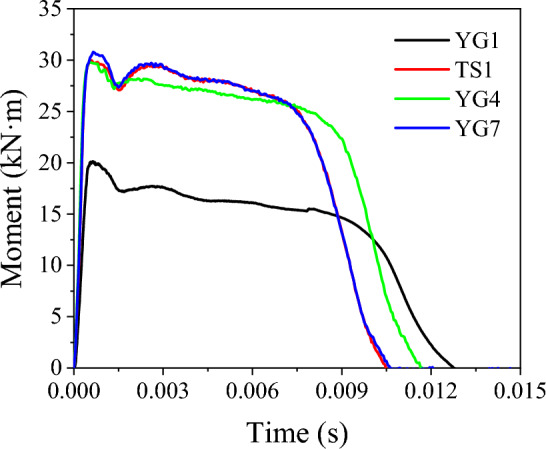
Impact point cross-section moment history from LS-DYNA.

The moment variation at the impact point of the specimens, caused by the lateral impact, is shown in Fig. 14. At the beginning of the impact, the moment increased sharply to the ultimate value in a very short time. Then, the moment decreased to a certain value and remained at this value for a relatively long time, covering the main period of the impact. This moment can be defined as the ultimate plastic moment capacity. The dynamic ultimate plastic moment capacity M_dp_, obtained from LS-DYNA, is presented in Table 10.

**Table 10:** Ultimate plastic moment capacity.

Specimen	*M*_p_ (kN·m)	^1^*M*_dp_ (kN·m)	^2^*M*_dp_ (kN·m)	*M*_dp_^2^/*M*_dp_^1^
YG1	10.8	16.0	17.5	1.09
TS1	16.4	27.7	26.5	0.96
YG4	16.4	26.7	26.9	1.01
YG7	16.4	27.7	26.5	0.96

## Proposed calculation method

### Ultimate plastic moment capacity

In this analysis, the ultimate plastic moment *M*_p_ is an important parameter. Elchalakani et al. presented a simplified rigid plastic approach for determining the ultimate plastic moment capacity of circular CFST members^52^, which can be expressed as follows:

7 $$M_{{\text{p}}} = \frac{2}{3}f_{{\text{c}}} \left( {\frac{D}{2} - h} \right)^{3} \cos^{3} \gamma_{0} \, + \,4f_{{\text{y}}} \left( {\frac{D - h}{2}} \right)^{2} h\cos \gamma_{0}$$

Here, γ_0_ represents the angular location of the plastic neutral axis. It can be calculated as

8 $$\gamma_{0} = \frac{{\frac{\pi }{{4}}\frac{{f_{{\text{c}}} \left( {D/2 - h} \right)^{2} }}{{f_{{\text{y}}} \left( {D/2 - h/2} \right)h}}}}{{2 + \frac{{f_{{\text{c}}} \left( {D/2 - h} \right)^{2} }}{{f_{{\text{y}}} \left( {D/2 - h/2} \right)h}}}}$$

*M*_p_ represents the static ultimate plastic moment. Based on *M*_p_, the dynamic ultimate plastic moment *M*_dp_ can be expressed as

9 $$M_{{{\text{dp}}}} = \lambda_{1} \frac{2}{3}f_{{\text{c}}} \left( {\frac{D}{2} - h} \right)^{3} \cos^{3} \gamma_{0} \, + \,4\lambda_{2} f_{{\text{y}}} \left( {\frac{D - h}{2}} \right)^{2} h\cos \gamma_{0}$$

10 $$\gamma_{0} = \frac{{\frac{\pi }{{4}}\frac{{\lambda_{1} f_{{\text{c}}} \left( {D/2 - h} \right)^{2} }}{{\lambda_{2} f_{{\text{y}}} \left( {D/2 - h/2} \right)h}}}}{{2 + \frac{{\lambda_{1} f_{{\text{c}}} \left( {D/2 - h} \right)^{2} }}{{\lambda_{2} f_{{\text{y}}} \left( {D/2 - h/2} \right)h}}}}$$

where *λ*_1_ and *λ*_2_ represent the DIFs for concrete and steel, respectively, at the rotation rate $\dot{\theta }$. DIFs *λ*_1_ and *λ*_2_ can be determined using Eqs. (1), (2), (3) when the rotation rate is $\dot{\theta }$. As discussed in Velocity variation at impact point, assuming that the lateral velocity of the impact point decreases linearly over time, the rate of rotation $\dot{\theta }$ of the mid-span section is defined as follows:

11 $$\dot{\theta } = \dot{\theta }_{1} + \dot{\theta }_{2} = \frac{{V_{{0}} }}{{2l_{1} }} + \frac{{V_{{0}} }}{{2l_{2} }}$$

Table 10 presents the static ultimate plastic moment *M*_p_ of the specimens (calculated using equations (7) and (8)) and the dynamic ultimate plastic moment (^1^*M*_dp_ was obtained from LS-DYNA, and ^2^*M*_dp_ was calculated using equations (9), (10) and (11)). The slight discrepancy between ^1^*M*_dp_ and ^2^*M*_dp_ confirms the accuracy of the calculation method.

### Three phases of motion

Jones^28^ theoretically derived the dynamic response of a beam under a mid-span impact with fixed–fixed supports. Wang^53^ has theoretically deduced the dynamic response of rigid-plastic structures subjected to lateral impact at an arbitrary point and divided their motion process into three phases. According to Wang’s research, a rigid-plastic model and the travelling plastic hinge theory were adopted to calculate the deflection of an arbitrary impact point on the CFST members in this study.

**Figure 15:**
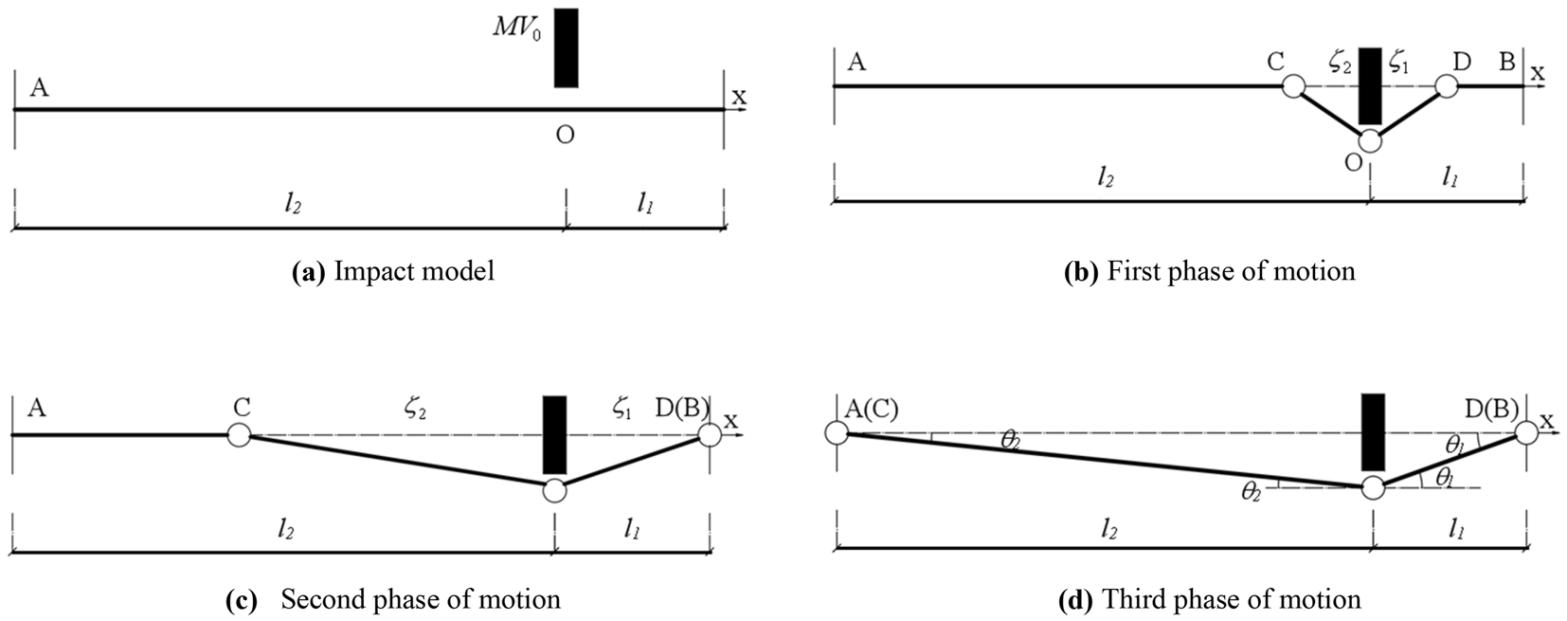
Impact process.

The basic mechanical model is shown in Fig. 15a. A member with a length of *l*_1_+*l*_2_ is under a lateral impact at an arbitrary point O by a mass *M* with an initial velocity *V*_0_. The impact point travels with a velocity *V*_0_ at the instant of impact while the rest of the member remains stationary. Therefore, the disturbance at the impact point propagates to both supports of the member. Then, global bending deformation occurs, dissipating the remaining impact energy, and finally ceases. This impact process can be divided into three distinct phases of motion, hereinafter referred to as the first, second, and third phases of motion. In this analysis, the influence of the axial load is neglected, and it is considered that the yield shear force of the cross-section is sufficiently large to prevent slippage.

#### First phase of motion

As shown in Fig. 15b, in the first phase of motion, a plastic hinge develops at the impact point at *t* = 0, and two other plastic hinges propagate the disturbance away from the impact point toward the two supports. Then, travelling plastic hinge D reaches support B, forming a stationary plastic hinge.

O is taken as the origin of the coordinate system, and the positive direction of the X-axis is to the right. The lateral velocity field at an arbitrary point of the member in the first phase of motion is expressed as follows:

12 $$\dot{w} = \left\{ \begin{gathered} \dot{w}_{0} \left( {1 + x/\zeta_{2} } \right),\,\left( { - \zeta_{2} \le x < 0} \right) \hfill \\ \dot{w}_{0} \left( {1 - x/\zeta_{{1}} } \right),\,\left( {{0} \le x < \zeta_{1} } \right) \hfill \\ 0,\left( { - l_{2} \le x < - \zeta_{2} ,\zeta_{1} \le x \le l_{{1}} } \right) \hfill \\ \end{gathered} \right.$$

where *ζ*_1_ and *ζ*_2_ represent the time-dependent locations of travelling plastic hinges D and C, respectively, and $\dot{w}_{0}$ represents the lateral velocity at the impact point.

Taking half of the member for analysis, the moments of impact point O are given as follows:

13 $${2}M_{{{\text{dp}}}} - \int_{0}^{{\zeta_{1} }} m \ddot{w}xdx = 0$$

14 $${2}M_{{{\text{dp}}}} - \int_{{ - \zeta_{2} }}^{0} m \ddot{w}xdx = 0$$

Combining equations (13) and (14) yields *ζ*_1_ = *ζ*_2_.

Because the lateral shear force is zero at the travelling plastic hinges where the maximum bending moment develops, the vertical equilibrium for the central portion of the member between the two travelling plastic hinges C and D demands that

15 $$M\ddot{w}_{0} + \int_{{ - \zeta_{2} }}^{{\zeta_{1} }} m \ddot{w}dx = 0$$

After Eq. (12) is substituted into Eq. (15), integrating Eq. (15) with respect to time and using the initial conditions $\dot{w}_{0}$ = 0 and *ζ*_1_ = 0 at *t* = 0 yields

16 $$\dot{w}_{0} = V_{0} /\left( {1 + m\zeta_{1} /M} \right)$$

Substituting equation (12) into equation (13) yields

17 $$\int_{0}^{{\zeta_{1} }} {m[\ddot{w}_{0} \left( {1 - x/\zeta_{1} } \right) + \dot{w}_{0} x\dot{\zeta }_{1} /\zeta_{1}^{2} ]} xdx = {2}M_{{{\text{dp}}}}$$

Which can be written in the following form:

18 $$d(\dot{w}_{0} \zeta_{1}^{2} )/dt = 12M_{{{\text{dp}}}} /m$$

Finally, integrating equation (18) with respect to time and using the initial conditions*ζ*_1_ = 0 at *t* = 0 yields the location-time characteristic of the travelling plastic hinge:

19 $$t = \frac{{MmV_{0} \zeta_{1}^{2} }}{{12M_{{{\text{dp}}}} \left( {M + m\zeta_{1} } \right)}}$$

Taking the derivative of equation (19) with respect to time predicts the velocity of the travelling plastic hinge:

20 $$\dot{\zeta }_{1} { = }\frac{{12M_{{{\text{dp}}}} (M + m\zeta_{1}^{2} )^{2} }}{{M{\text{m}}\zeta_{1} V_{0} \left( {2M + m\zeta_{1} } \right)}}$$

At the end of the first phase of motion (*t* = *t*_1_), the lateral deflection of the first phase of motion at the impact point *w*_01_ can be described through the following equation:

21 $$w_{01} = \int_{0}^{{t_{1} }} {\dot{w}_{0} } dt = \int_{0}^{{l_{1} }} {\dot{w}_{0} d\dot{\zeta }_{1} /\dot{\zeta }_{1} }$$

Substituting equations (16) and (20) into equation (21) yields

22 $$w_{01} = \frac{{M^{2} V_{0}^{2} }}{{24M_{{{\text{dp}}}} m}}\left[ {2\ln \left( {\frac{{ml_{1} + M}}{M}} \right) + \frac{{M^{2} }}{{\left( {M + ml_{1} } \right)^{2} }} - 1} \right]$$

#### Second phase of motion

As shown in Fig. 15c, in the second phase of motion, after plastic hinge D becomes a stationary hinge, plastic hinge C continues moving toward support A until it reach support A and forms a stationary plastic hinge.

In the second phase of motion, the bending moment at plastic hinge D may no longer be the extreme point in the bending-moment diagram; thus, there is a nonzero shear force *Q* at plastic hinge D. Additionally, *ζ*_1_ = *l*_1_. The lateral velocity field in the second phase of motion can be expressed as follows:

23 $$\dot{w}{ = }\left\{ \begin{gathered} \dot{w}_{0} \left( {1 + x/\zeta_{2} } \right),\,\left( { - \zeta_{2} \le x < 0} \right) \hfill \\ \dot{w}_{0} \left( {1 - x/l_{{1}} } \right),\,\left( {{0} \le x \le l_{1} } \right) \hfill \\ 0,\left( { - l_{2} \le x < - \zeta_{2} } \right) \hfill \\ \end{gathered} \right.$$

**Figure 16:**
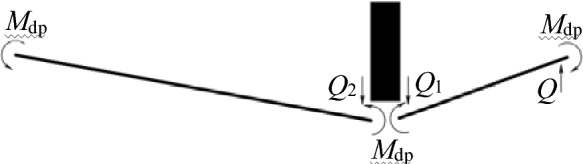
Force analysis diagram.

As shown in Fig. 16, the force balance can be expressed as:

24 $$\left\{ \begin{gathered} \frac{m}{2}(\zeta_{2} \ddot{w}_{0} { + }\dot{\zeta }_{2} \dot{w}_{0} ) = Q_{2} \hfill \\ \frac{m}{2}l_{1} \ddot{w}_{0} = Q_{1} - Q \hfill \\ \end{gathered} \right.$$

Where *Q*_*1*_ and *Q*_*2*_ represent the shear forces on the left and right sides of O, respectively. *Q* represents the shear force at plastic hinge D. The bending-moment balance can be expressed as:

25 $$\left\{ \begin{gathered} \frac{m}{12}\zeta_{2} \left( {\zeta_{2} \ddot{w}_{0} - \dot{\zeta }_{2} \dot{w}_{0} } \right) = \frac{1}{2}\zeta_{2} Q_{2} - 2M_{{{\text{dp}}}} \hfill \\ \frac{m}{12}l_{1}^{2} \ddot{w}_{0} = \frac{1}{2}l_{1} \left( {Q_{1} { + }Q} \right) - 2M_{{{\text{dp}}}} \hfill \\ \end{gathered} \right.$$

Combining equation (24) and equation (25) yields

26 $$\left( {\frac{1}{3}ml_{1}^{2} { + }Ml_{1} } \right)\ddot{w}_{0} + \frac{1}{2}ml_{1} \left( {\zeta_{2} \ddot{w}_{0} + \dot{\zeta }_{2} \dot{w}_{0} } \right) = - 2M_{{{\text{dp}}}}$$

27 $$m\zeta_{2} \left( {\zeta_{2} \mathop {w_{0} }\limits^{ \cdot \cdot } + 2\mathop {\zeta_{2} }\limits^{ \cdot } \mathop {w_{0} }\limits^{ \cdot } } \right) = 12M_{P}$$

Integrating equations (26) and (27) with respect to time and using the continuous conditions at *t*_1_ yield

28 $$\left( {\frac{1}{3}ml_{1}^{2} { + }Ml_{1} + \frac{1}{2}ml_{1} \zeta_{2} } \right)\dot{w}_{0} = - 2M_{{{\text{dp}}}} t + Ml_{1} V_{0}$$

29 $$m\zeta_{2}^{2} \dot{w}_{0} = 12M_{{{\text{dp}}}} t$$

By combining equations (28) and (29), the velocity at the impact point can be determined as follows:

30 $$\dot{w}_{0} = \frac{{6Ml_{1} V_{0} }}{{2ml_{1}^{2} + 6Ml_{1} + 3ml_{1} \zeta_{2} + m\zeta_{2}^{2} }}.$$

The velocity of the traveling plastic hinge can be predicted by substituting equation (30) into equation (29) and then integrating equation (29) with respect to time:

31 $$\dot{\zeta }_{2} { = }\frac{{2M_{{{\text{dp}}}} (2ml_{1}^{2} + 6Ml_{1} + 3ml_{1} \zeta_{2} + m\zeta_{2}^{2} )^{2} }}{{Mml_{1} V_{0} \left( {3ml_{1} \zeta_{2}^{2} + 4ml_{1}^{2} \zeta_{2} + 12Ml_{1} \zeta_{2} } \right)}}.$$

Integrating the velocity with respect to time yields

32 $$w_{02} = \int_{{t_{1} }}^{{t_{2} }} {\dot{w}_{0} } dt = \int_{{l_{1} }}^{{l_{2} }} {\dot{w}_{0} d\zeta_{2} /\dot{\zeta }_{2} } ,$$

where *w*_02_ represents the lateral deflection of the second phase at the impact location.

Substituting equations (30) and (31) into equation (32) yields

33 $$w_{02} = \frac{{3M^{2} ml_{1}^{2} V_{0}^{2} }}{{M_{{{\text{dp}}}} }}\int_{{l_{1} }}^{{l_{2} }} {\frac{{3ml_{1} \zeta_{2}^{2} + 4ml_{1}^{2} \zeta_{2} + 12Ml_{1} \zeta_{2} }}{{(2ml_{1}^{2} + 6Ml_{1} + 3ml_{1} \zeta_{2} + m\zeta_{2}^{2} )^{3} }}} d\zeta_{2} .$$

#### Third phase of motion

As shown in Fig. 15d, in the third phase of motion, the plastic hinges at the support and the impact location remain stationary, and the member continues moving downward under the action of the impact energy until the kinetic energy of the member and the impactor is exhausted. The third phase of motion is the final phase of the dynamic response of the CFST member under the impact. According to the rigid-plastic dynamic model, it is assumed that all the remaining kinetic energy of the impactor and member are consumed by plastic deformation in this phase.

According to motion analysis for the first and second phases of motion, as well as the kinetic energy theorem, the kinetic energy of span *l*_2_ and span *l*_1_ can be calculated as follows:

34 $$K_{2}^{{l_{2} }} = \int_{{ - l_{2} }}^{0} {\frac{1}{2}m\left[ {\frac{{6Ml_{1} V_{0} }}{{2ml_{1}^{2} + 6Ml_{1} + 3ml_{1} l_{2} + ml_{2}^{2} }}(1 + x/l_{2} )} \right]^{2} dx} { = }\frac{{6mM^{2} l_{1}^{2} l_{2} V_{0}^{2} }}{{(2ml_{1}^{2} + 6Ml_{1} + 3ml_{1} l_{2} + ml_{2}^{2} )^{2} }},$$

35 $$K_{2}^{{l_{1} }} = \int_{0}^{{l_{1} }} {\frac{1}{2}m\left[ {\frac{{6Ml_{1} V_{0} }}{{2ml_{1}^{2} + 6Ml_{1} + 3ml_{1} l_{2} + ml_{2}^{2} }}\left( {1 - x/l_{{1}} } \right)} \right]^{2} dx} { = }\frac{{6mM^{2} l_{1}^{3} V_{0}^{2} }}{{(2ml_{1}^{2} + 6Ml_{1} + 3ml_{1} l_{2} + ml_{2}^{2} )^{2} }}.$$

The kinetic energy of the impactor is given as follows:

36 $$K_{1} { = }\frac{1}{2}M\left[ {\frac{{6Ml_{1} V_{0} }}{{2ml_{1}^{2} + 6Ml_{1} + 3ml_{1} l_{2} + ml_{2}^{2} }}} \right]^{2} { = }\frac{{18M^{3} l_{1}^{2} V_{0}^{2} }}{{(2ml_{1}^{2} + 6Ml_{1} + 3ml_{1} l_{2} + ml_{2}^{2} )^{2} }}.$$

The total kinetic energy of the system is the sum of the kinetic-energy values of the member and the impactor:

37 $$K = K_{1} + K_{2}^{{l_{1} }} + K_{2}^{{l_{2} }} = \frac{{18M^{3} l_{1}^{2} V_{0}^{2} + 6mM^{2} l_{1}^{2} V_{0}^{2} (l_{1} + l_{2} )}}{{(2ml_{1}^{2} + 6Ml_{1} + 3ml_{1} l_{2} + ml_{2}^{2} )^{2} }}.$$

According to the assumption for the third phase of motion, the energy-balance principle, and the rigid-plastic model, all the kinetic energy of the system is dissipated by the plastic hinge. Then,

38 $${2}M_{{{\text{dp}}}} \theta_{1} + 2M_{{{\text{dp}}}} \theta_{2} = K$$

where* θ*_1_ and *θ*_2_ represent the angle of rotation of travelling plastic hinges D and C, respectively.

From the lateral deflection field and the geometric relationship of the member, the following equation can be obtained:

39 $$\theta_{{1}} l_{1} = \theta_{2} l_{2} .$$

By combining equations (37), (38), (39), the lateral deflection of the third phase at the impact location *w*_03_ can be calculated as follows:

40 $$w_{{{03}}} = \frac{{18M^{3} l_{1}^{3} l_{2} V_{0}^{2} + 6mM^{2} l_{1}^{3} l_{2} V_{0}^{2} (l_{1} + l_{2} )}}{{M_{{{\text{dp}}}} (2l_{1} + 2l_{2} )(2ml_{1}^{2} + 6Ml_{1} + 3ml_{1} l_{2} + ml_{2}^{2} )^{2} }}$$

By unifying the calculations and analyses for the three phases of motion, a theoretical deflection calculation equation for CFST members was obtained:

41 $$w_{0} = w_{01} + w_{02} + w_{03}$$

Using Equation (41), the deflection of the impact points of YG1, TS1, and YG4 under the lateral impact were calculated, and the results are presented in Table 11. The calculation errors of the method were <8%, which satisfies the accuracy requirements of engineering.

**Table 11:** Deflection of the members.

Specimen label	$\Delta$(mm)	$w_{0}$(mm)	Error
YG1	32.2	34.08	5.8%
TS1	34.8	37.53	7.9%
YG4	49.5	51.69	4.4%

### Error analysis

Based on the ideal rigidity-plasticity of materials and the theory of travelling plastic hinges, a method to calculate the deflection of CFST members at the impact point was developed. In the theoretical derivation, a simplified rigid-plastic model was applied, assuming that the plastic hinges dissipate entirely all the energy. Nevertheless, in the actual case where CFST members bear a lateral impact, the materials are not rigid-plastic as supposed; thus, not all the energy is absorbed by the members, as shown in Fig. 7 and Table 6. Furthermore, local compression deformation, steel tube buckling deformation at the edge of the support, and frictional energy dissipation at the restrained end can also consume energy, as shown in Fig. 4. Therefore, the energy during the impact process is not entirely dissipated by plastic deformation.

## Conclusions

The following conclusions are drawn based on the research presented in this paper:

1. Lateral non-mid-span impact tests of CFST specimens were performed. It was found that: ① the global deformation and flexural cracks indicated that the specimens underwent flexural failure; ② the maximum deflection of CFST columns appeared at the impact location under very low-elevation lateral impact loads; ③ the maximum deflection is an essential index for CFST members under lateral impacts, which is related to the impact velocity and the thickness of the steel tube; ④ the recovered and absorbed energy were calculated by integrating the force–displacement curve, and the absorbed energy ratio was > 90%.
2. A FEA was performed for further study, revealing the following. ① The axial load had no significant effect on the transverse deflection of the specimens. With an increase in the axial compression ratio, the deflection tended to decrease and then increase, but the axial load had little influence on the transverse deflection (within 10%). ② The deflection of the model and prototype conforms to the similarity criterion if the specimen is designed strictly according to the similarity criterion. Therefore, the size effect on the transverse deformation can be ignored (within 6%). ③ The bending moment at the impact point of the specimen increases sharply after impact. After fluctuation, it remains stable at a certain value, that is, the ultimate plastic moment of the specimen. ④ The lateral velocity at the impact point decreases linearly before the maximum deflection of the specimen is reached.
3. According to the travelling plastic hinge theory and the assumption of the ideal rigid-plastic model, the motion of CFST members subjected to lateral impact can be divided into three phases. Accordingly, a mechanical analysis of the CFST member during each phase was performed to calculate the deflection of the member and a corresponding theoretical method was proposed.

## Data Availability

The datasets used and analyzed during the current study are available from the corresponding author upon reasonable request.

## Abbreviations

*D*: Outer diameter of circular steel tube
*E*_c_: Modulus of elasticity of concrete
*Es*: Modulus of elasticity of steel
*F*: Axial load
F_o_: Axially compressive capacity
*f*_c_: Cylinder compressive strength of concrete
*f*_cd_: Dynamic compressive strength of concrete
*f*_ck_: Characteristic concrete strength
*f*_cs_: Static compressive strength of concrete
*f*_cu_: Cube compressive strength of concrete
*f*_td_: Dynamic tensile strength of concrete
*f*_ts_: Static tensile strength of concrete
*f*_u_: Ultimate strength of steel
*f*_y_: Yield strength of steel
*f*_yd_: Dynamic yield strength of steel
*f*_ys_: Static yield strength of steel
*γ*_0_: Angular location of the plastic neutral axis
*η*: Geometric scale factor
*K*: Kinetic energy
*L*: Clear span of members
*l*_1_: Distance from impact point to the proximal support
*l*_2_: Distance from impact point to the distal support
*m*: Mass per unit length of members
*M*: Impactor mass
*M*_dp_: Dynamic ultimate plastic moment
*M*_p_: Static ultimate plastic moment
*M*_s_: Moment
*Mu*: Moment capacity
*n*: Axial load level (= *F*/*F*_u_)
*Q*: Shear force
*θ*: Rotation degree
*S*: Impact surface area
*t*_*s*_: Thickness of steel tube
*V*_0_: Initiation impact velocity
*w*_0_: Predicted maximum deflection at impact point
*ζ*: Time-dependent location of plastic hinges
Δ: Test maximum deflection at impact point
*λ*_1_: Dynamic increase factor for concrete
*λ*_2_: Dynamic increase factor for steel
*μ*_c_: Poisson's ratio of concrete
*μ*_s_: Poisson's ratio of steel
*ρ*_c_: Density of concrete
*ρ*_I_: Density of impactor
*ρ*_s_: Density of steel
$$\dot{\varepsilon }$$: Strain rate
$$\dot{\theta }$$: Rotation rate
$\dot{w}$: Lateral velocity at impact point
$\ddot{w}$: Lateral accelerate at impact point
